# Effect of lateral septum vasopressin administration on reward system neurochemistry and amphetamine-induced addictive-like behaviors in female rats

**DOI:** 10.3389/fphar.2024.1411927

**Published:** 2024-07-29

**Authors:** Macarena Francisca Gárate-Pérez, Daniela Cáceres-Vergara, Francisca Tobar, Carolina Bahamondes, Tamara Bahamonde, Claudia Sanhueza, Fanny Guzmán, Ramón Sotomayor-Zárate, Georgina M. Renard

**Affiliations:** ^1^ Universidad de Santiago de Chile (USACH), Facultad de Ciencias Médicas, Escuela de Medicina, Centro de Investigación Biomédica y Aplicada (CIBAP), Santiago, Chile; ^2^ Centro de Neurobiología y Fisiopatología Integrativa (CENFI), Instituto de Fisiología, Facultad de Ciencias, Universidad de Valparaíso, Valparaíso, Chile; ^3^ Laboratorio de Síntesis de Péptidos, Núcleo de Biotecnología Curauma (NBC), Pontificia Universidad Católica de Valparaíso, Valparaíso, Chile

**Keywords:** lateral septum, vasopressin, amphetamine, sex differences, GABA, glutamate, dopamine

## Abstract

**Introduction:** The chronic use of psychostimulants increases the risk of addiction and, there is no specific pharmacologic treatment for psychostimulant addiction. The vasopressin (AVP) system is a possible pharmacological target in drug addiction. Previous results obtained in our laboratory showed that amphetamine (AMPH) treatment decreases lateral septum (LS) AVP levels in male rats, and AVP microinjection in LS decreases addictive-like behavior. The aim of the present work was to investigate the effect of AMPH treatment on LS AVP levels and the effect of LS AVP administration on the expression of AMPH-conditioned place preference (CPP) in female rats. The secondary objectives were to study the effect of LS AVP administration on LS GABA and glutamate release in male and female rats and on nucleus accumbens (NAc) dopamine (DA) release in female rats.

**Methods:** Female rats were conditioned with AMPH (1.5 mg/kg i.p.) or saline for 4 days.

**Results:** Conditioning with AMPH did not change LS AVP content in females. However, AVP microinjection into the LS decreased the expression of conditioned place preference (CPP) to AMPH. Glutamate and GABA extracellular levels in the LS induced by AVP were studied in males and females. NAc GABA and DA extracellular levels induced by LS AVP microinjection in female rats were measured by microdialysis. In males, AVP perfusion produced a significant increase in LS GABA extracellular levels; however, a decrease in GABA extracellular levels was observed in females. Both in males and females, LS AVP perfusion did not produce changes in LS glutamate extracellular levels. Microinjection of AVP into the LS did not change GABA or DA extracellular levels in the NAc of females.

**Discussion:** Therefore, AVP administration into the LS produces different LS-NAc neurochemical responses in females than males but decreases CPP to AMPH in both sexes. The behavioral response in males is due to a decrease in NAc DA levels, but in females, it could be due to a preventive increase in NAc DA levels. It is reasonable to postulate that, in females, the decrease in conditioning produced by AVP microinjection is influenced by other factors inherent to sex, and an effect on anxiety cannot be discarded.

## 1 Introduction

There is a great use and abuse of psychostimulants around the world, and amphetamines are the second most used psychostimulant (Ronsley et al., 2020). The chronic use of psychostimulants increases the risk of addiction, which is a chronic brain disease characterized by the compulsive use of the drug and the emergence of a negative emotional state during abstinence (Koob and Volkow, 2010; Volkow et al., 2019). Moreover, it has been shown that the addictive process presents sex differences; women develop a substance use disorder more quickly after the first consumption and with low doses, and usually, women have worse abstinence effects than men (Becker et al., 2017). At present, there is no specific pharmacologic treatment for psychostimulant addiction, and behavioral therapy is still a unique therapy with limited evidence about its efficacy (Ronsley et al., 2020). Several years ago, in the search for a treatment, neuropeptide systems began to be studied as a possible pharmacological target in drug addiction (DiLeone et al., 2003; Cid-Jofré et al., 2021). One is vasopressin (AVP), a neuropeptide that presents anatomic and functional sex differences in rodents, regulates neurotransmitter release, and modulates reward system function (Allaman-Exertier et al., 2007; de Vries, 2008; Bredewold et al., 2018; Gárate-Pérez et al., 2021).

The reward system involves dopaminergic projections from the ventral tegmental area (VTA) to the nucleus accumbens (NAc) and prefrontal cortex (PFC) (Koob and Volkow, 2010; Volkow et al., 2019). However, other nuclei, such as the lateral septum (LS), are involved in its regulation. The LS is a brain nucleus implicated in several behaviors, such as social behaviors and addiction processes. It received vasopressinergic projection from the bed nucleus of the stria terminalis (BNST) and medial amygdala (MeA). These projections are sexually dimorphic, being more pronounced in males (de Vries et al., 1983; de Vries, 2008; Rigney et al., 2023), who expressed higher levels of the V_1A_ AVP receptor than females in some areas such as BNST, somatosensory cortex, piriform cortex, lateral hypothalamus among others (Dumais and Veenema, 2016). The AVP system has been implicated in drug use and addiction (Godino and Renard, 2018; Perry and Cornish, 2022). It has been shown that acute AMPH administration and AMPH sensitization increased AVP mRNA levels in the MeA and BNST, respectively, only in females (Ahumada et al., 2017). Regarding vasopressin receptors, activation of the V_1B_ receptor is proposed to contribute to addiction because of the activation of the hypothalamic-pituitary-adrenal axis and its antagonism reduces addictive behaviors (Godino and Renard, 2018; Perry and Cornish, 2022). Conversely, activation of the V_1A_ receptor has been primarily associated with a reduction in addictive behaviors. It has been shown that V_1A_ activation reduces methamphetamine-primed reinstatement (Everett et al., 2018); however, NAc V_1A_ activation increases the expression of cocaine conditioning (Rodriguez-Borrero et al., 2010).

On the other hand, it has been described a circuitry in which GABAergic projections from LS to VTA inhibit GABA interneurons in VTA, increasing dopamine (DA) release (Luo et al., 2011; Vega-Quiroga et al., 2018). Interestingly, it has been shown by electrophysiological studies that AVP increased the GABAergic inhibitory tone in the LS by activating the V_1A_ receptor, suggesting an inhibition of GABAergic projections from the LS (Raggenbass, 2008). Regarding functional sex differences, the administration of AVP in the LS improved social recognition only in juvenile females, and the V_1A_ antagonist increases social play behavior in juvenile males and decreases this behavior in females (Bredewold and Veenema, 2018). Previous results obtained in our laboratory showed that AMPH treatment decreased LS AVP levels in male rats, and AVP microinjection in the LS decreased addictive-like behavior, decreasing NAc DA levels (Gárate-Pérez et al., 2021).

The primary objective of the present work was to investigate the effect of AMPH treatment on LS AVP levels and the effect of LS AVP administration on the expression of AMPH-conditioned place preference in female rats to evaluate possible sex differences. The secondary objectives were to study the effect of LS AVP administration on i) LS GABA and glutamate release in male and female rats and ii) NAc DA release after AMPH-induced CPP in female rats.

## 2 Materials and methods

### 2.1 Reagents

[Arg8]-Vasopressin (AVP) was synthesized according to previously described methods (Luna et al., 2016) at the Núcleo de Biotecnología Curauma, Pontificia Universidad Católica de Valparaíso. Amphetamine sulfate was donated by Laboratorio Chile S.A. . (d (CH2)51, Tyr (Me)2, Arg8)-Vasopressin (V_1A_ Ant), a selective antagonist for V_1A_ receptors, was purchased from Tocris Bioscience. All other reagents were of analytical and molecular grade.

### 2.2 Animals

Seven male and 84 female *Sprague Dawley* rats (55–60 days old; 200–250 g) from the vivarium of the Universidad de Valparaíso or the Pontificia Universidad Católica de Chile (UC CINBIOT Animal Facility funded by PIA CONICYT ECM-07) were used. Animals were housed in a temperature and humidity-controlled room (22°C ± 2°C; 50% ± 5%) under artificial illumination (12-h light/12-h dark; light on at 08:00 a.m.). Water and food (5P00 Prolab^®^ RMH 3000) were given *ad libitum*. Experiments were performed according to protocols approved by the Bioethical and Biosecurity Committees of the Universidad de Santiago de Chile (N° 595–2017) and Universidad de Valparaíso (N° 027–2014). They were under the National Institutes of Health Guide for Care and Use of Laboratory Animals. All efforts were made to minimize animal suffering and to reduce the number of animals used.

### 2.3 Amphetamine conditioned place preference (CPP)

**FIGURE 1:**
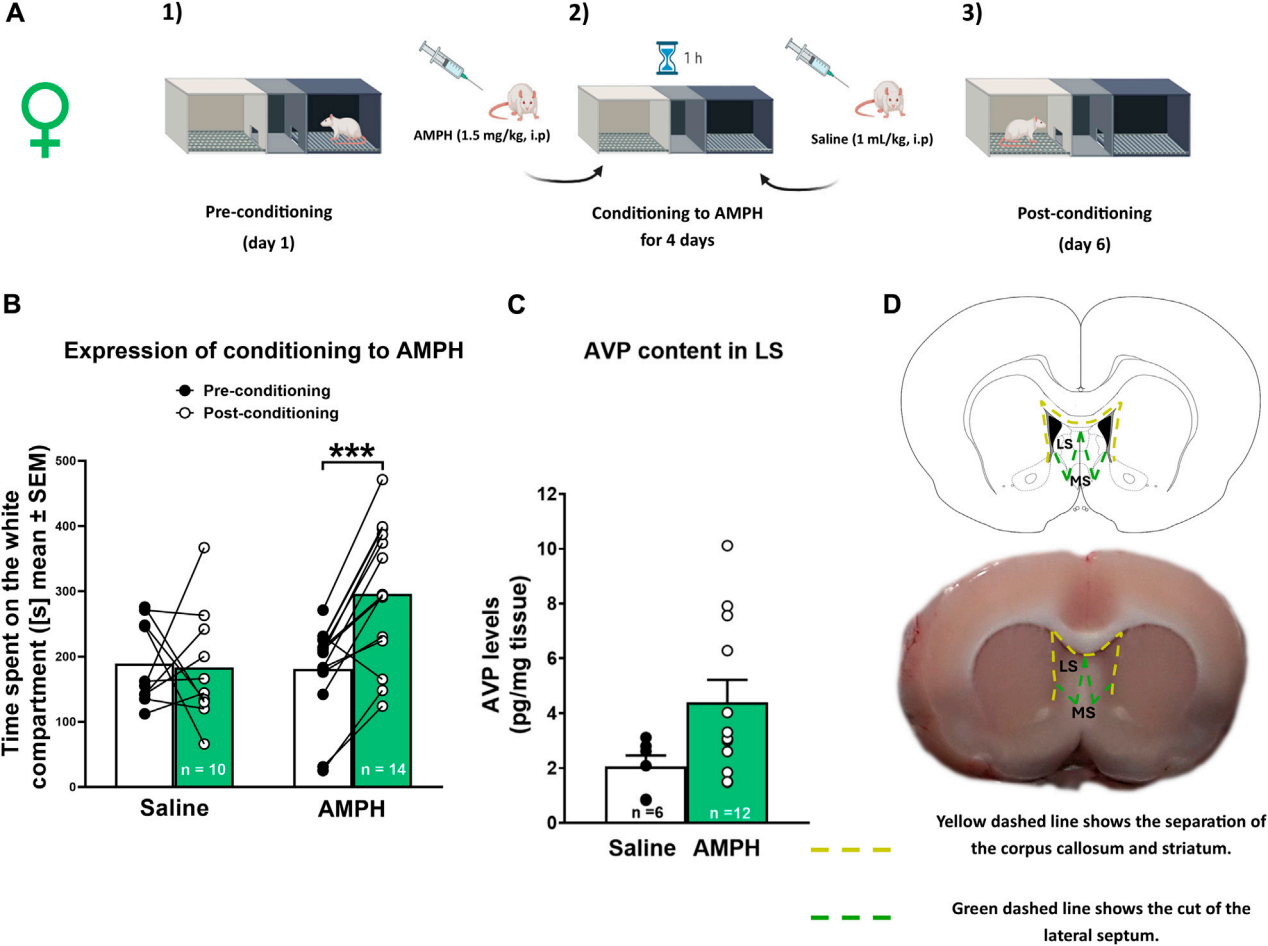
AMPH conditioned place preference and AVP tissue levels in female rats **(A)** Schematic representation of the CPP protocol. **(B)** Amphetamine (AMPH) treatment (1.5 mg/kg, i p.) elicited conditioned place preference (CPP) in female rats. Data are expressed as time spent on the white compartment [seconds (s), mean ± S.E.M] during the pre-and post-conditioning phase (saline n = 10; AMPH n = 14). ***p < 0.001 **(C)** Vasopressin AVP tissue content in the lateral septum (LS) of saline and AMPH-treated female rats. Data are expressed as pg/mg of tissue (mean ± S.E.M.; saline n = 6; AMPH n = 12). **(D)** Schematic representation of the LS dissection protocol.

A conditioning protocol adapted from Kotlinska et al. (2012) was used. AMPH was dissolved in 0.9% saline solution. The CPP apparatus consisted of three compartments: i) a white chamber, ii) a neutral corridor, and iii) a black chamber. The compartments were illuminated with 20 lux in the white chamber, ≤5 lux in the neutral corridor, and ≤1 lux in the black chamber. The CPP protocol consisted of three phases, as described in Gárate-Pérez et al. (2021) (Figure 1A y B) briefly: 1) A pre-conditioning phase in which the animal was allowed to explore all compartments freely for 15 min; 2) The conditioning phase in which animals received a daily dose of AMPH (1.5 mg/kg, i.p.) for 4 days in the afternoon and were immediately confined for 60 min in the compartment of basal lower preference (usually the white chamber). In the morning, animals received an injection of saline solution (1 mL/kg, i.p.) and were immediately confined for 60 min in the black compartment. Control animals received an injection of saline solution (1 mL/kg, i.p.) in the morning and the afternoon; 3) The post-conditioning phase, which was performed 24 h after conditioning and consisted of a 15-min test without AMPH, in which the animals were allowed to explore the entire apparatus. The time animals spent in the compartment associated with the drug was an index of its reinforcing value. A significant increase in the time spent on the white side in the post-conditioning phase indicated that the animals were conditioned to AMPH. Animals that spent more than 300 sec on the white side in the preconditioning phase were discarded from the analysis (n = 7; 3 in experiment 1 and 4 in experiment 2). The video tracking analysis was performed with ANY-Maze™ software (Stoelting Co., Illinois, United States) by an experimenter unaware of group assignments.

### 2.4 Surgeries

#### 2.4.1 Intra-LS cannulation

**FIGURE 2:**
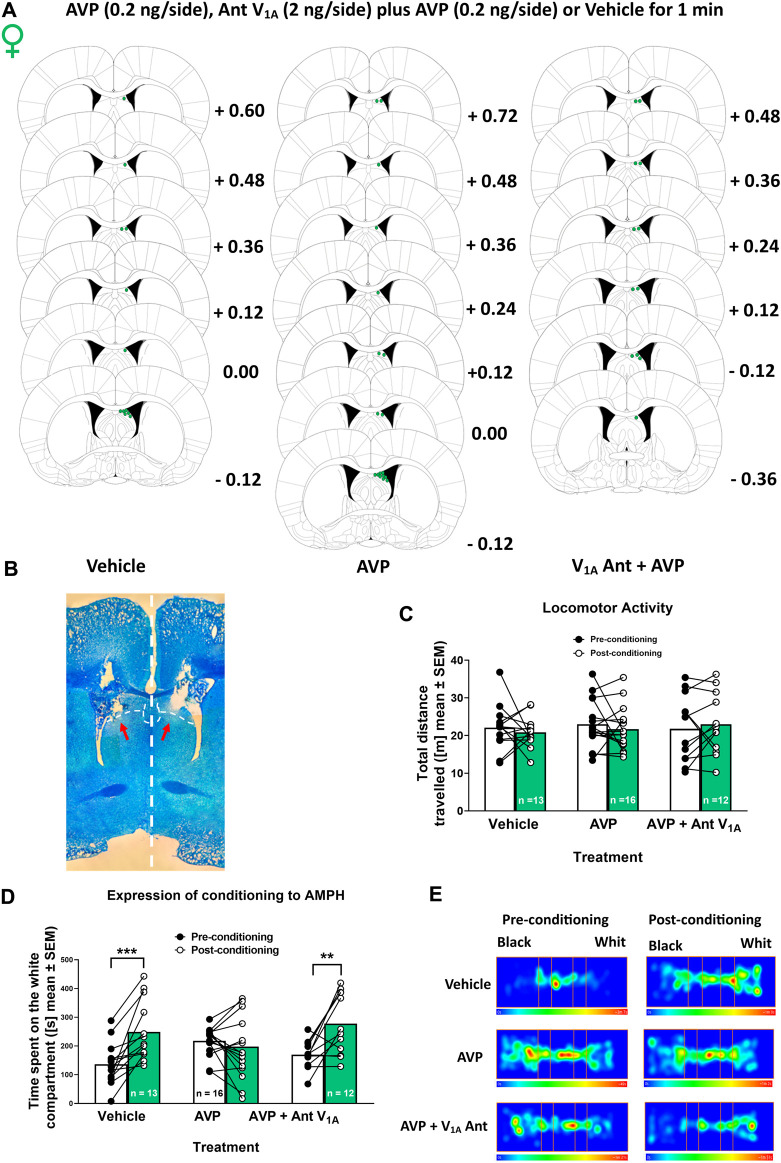
Effect of vasopressin (AVP) microinjection into lateral septum (LS) on locomotor activity and the expression of AMPH conditioned place preference in female rats **(A)** Microinjection placements in the LS of vehicle, AVP, and AVP plus V_1A_ antagonist female rats. Schematic representation of coronal brain sections [from Paxinos and Watson (2009)] indicates the correct placement of microinjection in the LS in green. Distance from Bregma is indicated on the right of schematic representations **(B)** Photomicrograph of a typical internal cannula placement into the LS. **(C)** Total distance traveled in the pre-conditioning phase and after vehicle, AVP or AVP plus V_1A_ antagonist microinjection in the post-conditioning phase. Data are expressed as meters (m; mean ± S.E.M.). **(D)** Time spent on the white compartment (seconds (s); mean ± S.E.M) during the pre-and post-conditioning phase of vehicle, AVP, and AVP + V_1A_ antagonist microinjected females. **p < 0.01; ***p < 0.001 (vehicle n = 13; AVP n = 16; AVP + V_1A_ ant n = 12). **(E)** Representative heat maps (one animal in each condition) denoting the time spent in the three compartments of the CPP apparatus after the different treatments, during the pre-conditioning and post-conditioning phases.

Animals were deeply anesthetized with isoflurane (Vaporizer Drager model 19.3, Germany, and pump air, model HAILEA ACO-208, China). 3% isoflurane and 1.5 mL/min of air for 10 min were used for induction. For maintenance, 1.2% isoflurane and 1.5 mL/min air during surgery were used, and lidocaine (6 mg/kg, s.c.) was injected in the implant area. Rats were placed in a stereotaxic apparatus (Model 68002, RWD Life Science Co., Ltd., China), and the incision area was cleaned with 2% chlorhexidine gluconate solution. Guide cannulas (Length 20 mm, Outer diameter 0.64 mm and internal diameter 0.45 mm; N° 62001, RWD Life Science Co., Ltd., Shenzhen, Guangdong, China) were implanted bilaterally in LS (angle 10°, + 0.1 mm anterior, ± 1.7 mm lateral and -2.3 mm ventral to bregma) using the atlas of Paxinos and Watson (2009) (Figure 2A). The microinjections were performed in the dorsal LS according to previous work in males (Gárate-Pérez et al., 2021) and because the circuitry from the hippocampus to the VTA through the LS involves the dorsal LS (Luo et al., 2011). The guide cannulas were fixed to the skull with stainless steel screws and a mixture of self-curing monomer with acrylic No. 66 (Duralay^®^, United States). During and after the surgery, the animals were kept on a heating pad until they came out of anesthesia. Rats were maintained for 6–7 days in recovery, and an analgesic (meloxicam 2 mg/kg, i. p) and antibiotic (enrofloxacin 5 mg/kg, p. o.) were administered for 3 days. During the post-surgery period, a monitoring protocol was followed to assess the recovery of the animals. This protocol included evaluating several parameters such as weight loss, coat condition, spontaneous behavior, and responses to stimuli.

#### 2.4.2 *In vivo* microdialysis

Rats were deeply anesthetized with isoflurane (Vaporizer Drager model 19.3, Germany, and pump air, model HAILEA ACO-208, China). Animals were anesthetized for surgery and during the microdialysis procedure, using 3% isoflurane and 1.5 mL/min of air for 10 min were used for induction. During microdialysis, maintenance was done in 1.5% isoflurane and 1.5 mL/min air for males and in 1.2% isoflurane and 1.5 mL/min air for females. The animals were placed in a stereotaxic apparatus, and their body temperature was maintained at 37°C with an electric blanket controlled by a thermostat. According to the experiment, a concentric cerebral microdialysis probe (Microdialysis Probe, Stockholm, Sweden; CMA-12, 20,000 Da limit, membrane length 2 mm, membrane diameter 0.5 mm) was implanted in the right LS or NAc. Microdialysis probes were perfused with aCSF at a 2 μL/min flow rate using an infusion pump (Model 210 RWD, RWD Life Science Co., Ltd., China). After a stabilization period of 90 min, when similar neurotransmitter concentrations were observed between samples, baseline perfusion samples were recollected (the percentage of variability between collected samples accepted is 10%). The protocol used is according to each experiment. At the end of each experiment, animals were sacrificed, and brains were quickly removed and stored in 4% PFA. Brain sections of 50 μm were stained with methylene blue, and the cannula or probe placement was examined microscopically.

### 2.5 Experiment 1: LS AVP tissue levels in females conditioning to AMPH

Female *Sprague Dawley* rats (n = 24 + n = 3 discarded from the analysis, see section 2.3) were used to study the effect of AMPH conditioning on LS AVP tissue content. The animals were obtained from the vivarium of the Faculty of Science of the Universidad de Valparaíso. Animals were randomly separated into two groups and were subjected to CPP: a) saline rats, injection of physiological saline solution (1.0 mL/kg, ip) during the conditioning period (4 days), and b) AMPH rats, injection of AMPH (1.5 mg/kg, ip, mL/kg) during the conditioning period (4 days).

#### 2.5.1 Tissue extraction and vasopressin tissue levels

One hour after the end of the test phase, rats were decapitated with a guillotine for small animals (model 51330**,** Stoelting Co., United States), and their brains were removed. With a brain matrix, a 2 mm brain section was obtained in which the LS (Bregma +1.6 to −0.4 approximately) was located according to the atlas of Paxinos and Watson (2009). The LS was then microdissected at 4°C using a scalpel. The LS was separated from the corpus callosum and the striatum through the lateral ventricles, and then a diagonal cut was made to eliminate the medial septum (Figure 1D). The dissected LS tissue was weighed on an analytical balance and stored at −80°C for further analysis. LS tissue samples were processed as described in Gárate-Pérez et al., 2021. Finally, LS AVP content was measured using a commercial AVP ELISA kit (Arginine Vasopressin EIA KIT No. 583951, Cayman Chemical, Ann Arbor, Michigan, United States) according to the manufacturer’s instructions, declaring that intra-assay variation is 5.9% and inter-assay variation is 6.4%.

### 2.6 Experiment 2: effects of AVP microinjection in the LS on the expression of conditioning to AMPH in female rats

#### 2.6.1 Microinjection protocol

Female rats (n = 41 + n = 4 discarded from the analysis; see Section 2.3) were obtained from the Pontificia Universidad Católica de Chile vivarium. After recovery, cannulated rats were subjected to the AMPH CPP protocol (i.e., all rats were treated with AMPH), as described above. 2 min before the post-conditioning phase of the CPP, two internal cannulas (Length beyond the guide cannula tip: 1 mm, N° 62201, RWD Life Science Co., Ltd., Shenzhen, Guangdong, China) were inserted bilaterally through a guide cannula. Each microinjection cannula was connected with FEP tubing to a syringe in an automated microperfusion pump (Model 210 RWD, RWD Life Science Co., Ltd., China) at 0.5 μL/min flow rate. Rats were divided into three experimental groups microinjected (during 1 min) bilaterally in LS (Figures 2A, B) with: 1) vehicle (artificial cerebrospinal fluid solution (aCSF), containing: 2.7 mM KCl, 147 mM NaCl, 0.85 mM MgCl_2_ and 1.2 mM CaCl_2_, at pH 7.4); 2) AVP in aCSF (0.4 ng/μL), and 3) AVP (0.4 ng/μL) plus V_1A_ Ant (4 ng/μL) in aCSF. The volume injected was 0.5 μL/side. The antagonist concentration used was ten times higher than the agonist concentration to avoid possible competition since we applied both drugs at the same time. Once the microinjection was finished, the microinjection cannulas were removed after 1 min to prevent the solutions from being returned by the capillary. Then, the post-conditioning phase of the CPP started. Rats were handled daily between surgery and CPP to familiarize them with the handling during the microinjection process.

### 2.7 Experiment 3: effects of AVP on LS GABA and glutamate release in male and female rats

#### 2.7.1 *In vivo* microdialysis and analysis of dialysate samples

**FIGURE 3:**
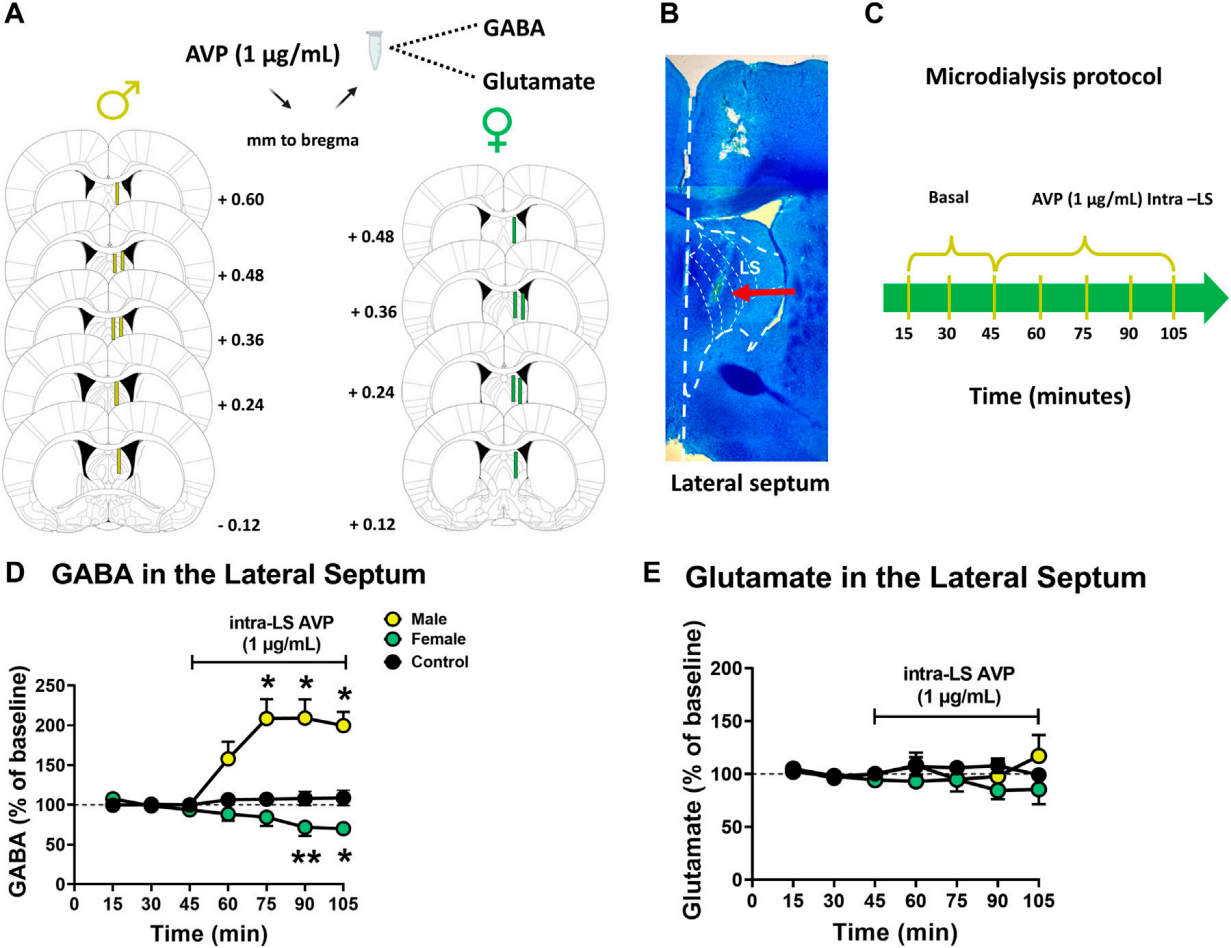
Effect of vasopressin (AVP) perfusion into lateral septum (LS) on GABA and glutamate extracellular levels in male and female rats **(A)** Microdialysis probe placement in the LS of male and female rats. Schematic representation of coronal brain sections [from Paxinos and Watson (2009)] indicates the correct probe placement in the LS in green. Distance from Bregma is indicated on schematic representations’ right (males) or left (females) **(B)** Photomicrograph of a typical microdialysis probe placement into the LS. **(C)** Schematic representation of the microdialysis protocol **(D)** GABA extracellular levels in the LS of male (yellow) and female (green) rats after AVP perfusion intra-LS and in the LS of a control male rats group (black). Results are expressed as a percentage of baseline ( ± S.E.M.) *p < 0.05; **p < 0.01 (males n = 7; females n = 6; control males n = 3). **(E)** Glutamate extracellular levels in the LS of male (yellow) and female (green) rats after AVP perfusion intra-LS and in the LS of a control male rats group (black). Results are expressed as a percentage of baseline ( ± S.E.M.) (males n = 7; females n = 6; control males n = 3).

Naïve male (n = 10) and female (n = 6) rats were obtained from the Pontificia Universidad Católica de Chile vivarium. The following coordinates were used according to the Paxinos and Watson (2009) atlas: AP, +0.2 mm; ML, + 0.7 mm; DV, - 5.2 mm for males and females LS, referred to bregma (Figures 3A, B). Three baseline perfusion samples (15, 30, and 45) of 15 min each were collected in 3 µL of 0.2 M perchloric acid (PCA). After collecting the third sample, an AVP solution (1 μg/mL) (Bosch and Neumann, 2010) was perfused into the LS for 60 min (Figure 3C). In the control group (n = 3 males), after collecting the third sample, another aCSF solution was perfused. The collected perfusion samples were stored at −80°C until analysis. At the end of each experiment, the animals were euthanized, and the brains were rapidly removed and stored in 4% paraformaldehyde (PFA).

#### 2.7.2 GABA and glutamate analysis

HPLC-fluorometric determination of GABA and glutamate was performed as described previously (Gárate-Pérez et al., 2021). Briefly, 20 μL of dialysis perfusate was mixed with 4 μL of borate buffer (pH 10.8). Then, the mixture was derivatized by adding 4 μL of fluorogenic reagent (20 mg of orthophthaldehyde and 10 μL of β-mercaptoethanol in 5 mL of ethanol). 90 s after derivatization, samples were injected into an HPLC system with the following configuration: isocratic pump (model PU-4180, Jasco Co., Ltd., Tokyo, Japan), a C-18 reverse phase column (Kromasil 3–4.6, Sweden), and fluorescence detector (model FP-4025, Jasco Co., Ltd., Tokyo, Japan). The mobile phase containing 0.1 M NaH_2_PO_4_ and 24.0% (^v^/_v_) CH_3_CN (pH adjusted to 5.7) was pumped at a 0.8 mL/min flow rate. The retention time for glutamate and GABA were 2.5 min and 18 min, respectively. Dialysate samples were analyzed by comparing the peak area and elution time with reference standards (ChromNAV 2.0, Jasco Co., Ltd., Tokyo, Japan).

### 2.8 Experiment 4: effects of AVP microinjection in the lateral septum on DA extracellular levels in the nucleus accumbens in female rats

**FIGURE 4:**
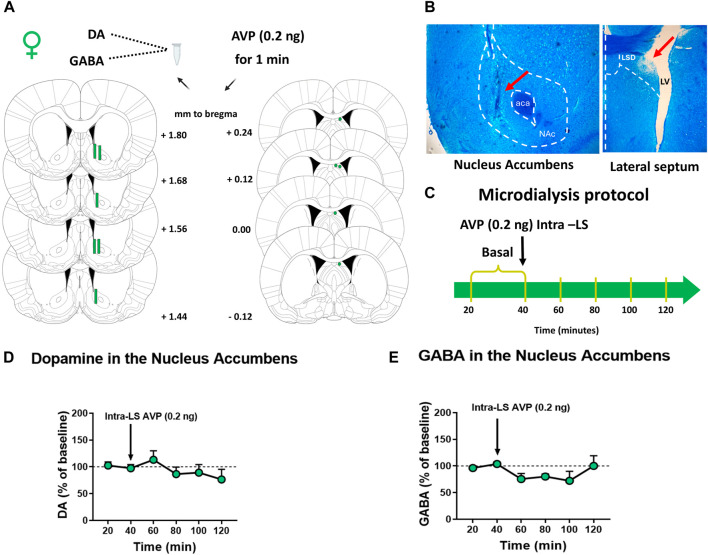
Effect of vasopressin (AVP) microinjection into the lateral septum (LS) on nucleus accumbens (NAc) DA and GABA extracellular levels of female rats subjected to AMPH-CPP **(A)** Microdialysis probe placement in the NAc and microinjection cannula placement in the LS of female rats. Schematic representation of coronal brain sections [from Paxinos and Watson (2009)] indicates the correct probe placement in the NAc or cannula placement in the LS (in green). Distance from Bregma is indicated on schematic representations’ right (NAc) or left (LS) **(B)** Photomicrograph of a typical microdialysis probe placement into the NAc and a typical internal cannula placement into the LS. **(C)** A schematic representation of the microdialysis protocol used **(D)** DA extracellular levels in the NAc of female rats after AVP microinjection intra-LS. Results are expressed as a percentage of baseline ( ± S.E.M.) (n = 5) **(E)** GABA extracellular levels in the NAc of female rats after AVP microinjection intra-LS. Results are expressed as a percentage of baseline ( ± S.E.M.) (n = 6).

Before microdialysis, the animals (n = 6) were subjected to AMPH CPP (all rats were treated with AMPH), as described before. The animals were obtained from the Pontificia Universidad Católica de Chile vivarium. After the post-conditioning phase, *in vivo* microdialysis in anesthetized animals was performed. Female rats were deeply anesthetized, as described before. Unilateral implantation of a guide cannula (23G/M 3.5, RWD) was performed on the right LS and fixed to the skull as described before. The following coordinates were used according to the atlas of Paxinos and Watson (2009): insertion angle of 10; AP: +0.1 mm, ML: +1.7 mm, DV: 2.3 mm, referring to bregma (Figures 4A, B). Then, a concentric brain microdialysis probe (Microdialysis Probe, Stockholm, Sweden; CMA-12, 20,000 Da cutoff, membrane length 2 mm, membrane diameter 0.5 mm) was implanted in the NAc using the following coordinates according to the atlas of Paxinos and Watson (2009) AP: +1.5 mm, ML: +1.5 mm, DV: 7.8 mm respect to bregma (Figures 4A, B). The microinjections and microdialysis were performed ipsilaterally according to LS-NAc connections (Deng et al., 2019). Two basal perfusion samples (20 and 40) of 20 min each were collected in 5 μL of 0.2 M PCA. After collecting basal 2, an AVP solution (0.4 ng/μL; 0.5 μL) was microinjected in the LS for 1 min by an infusion pump at 0.5 μL/min flow rate. Meanwhile, the collection of samples continued every 20 min in the NAc, between 40 and 120 min (Figure 4C). Collected perfusion samples were stored at −80°C until analysis. The dialysate samples were subsequently analyzed in the HPLC coupled to a fluorometric detector for GABA quantification described above and in an HPLC coupled to an electrochemical detector for DA quantification. Because DA levels could change depending on the estrus cycle, the animals subjected to microdialysis to measure DA levels in the NAc were estrous and proestrus, stages in which estradiol levels are higher than diestrus (Sharma et al., 2020).

#### 2.8.1 Dopamine analysis

HPLC-electrochemical determination of DA was performed as described previously (Gárate-Pérez et al., 2021). Briefly, 20 μL of dialysates were injected into an HPLC system with the following configuration: quaternary pump (model PU-2089s Plus), Jasco Co., Ltd., Tokyo, Japan), C-18 reverse phase column (Kromasil, Sweden), and amperometric detector (set at 750 mV, model ECD-700, EICOM). The mobile phase containing 0.15 M C_6_H_8_.7H_2_O, 0.8 mM C_8_H_17_NaO_4_S, 3% tetrahydrofuran, and 2.5% (^v^/_v_) CH_3_CN (pH adjusted to 3.0) was pumped at a flow rate of 0.2 mL/min. The retention time for DA was 8.5 min, and the detection limit was 0.1 fmol/μL. Dialysate samples were analyzed by comparing the peak area and elution time with reference standards (ChromNAV 2.0, Jasco Co., Ltd., Tokyo, Japan).

### 2.9 Statistics

Mann-Whitney test was used to determine significant differences in AVP levels in the LS between groups. Two-way ANOVA for repeated measures (RM) followed by Sidak *post hoc* test was used to determine significant differences in conditioning to AMPH and locomotor activity between the different groups. Friedman test for RM, followed by Dunn´s *post hoc* test, was used to determine significant differences between basal and post-AVP microdialysate samples in LS. One-way ANOVA for repeated measures (RM) followed by Sidak *post hoc* test was used to determine significant differences between basal and post-LS AVP microinjection in the NAc DA extracellular levels. The F was used to compare two variances, and the Bartlett test was conducted to assess the homogeneity of variance. In cases where this assumption was not met, non-parametric statistics were employed. Significance was set at *p* < 0.05. The statistical analysis was done in GraphPad Prism v7.0 (GraphPad Software, San Diego, CA).

## 3 Results

### 3.1 Experiment 1: LS AVP tissue levels in females conditioning to AMPH

AVP levels were determined in the LS of females after conditioning to AMPH. Figure 1B shows the time spent on the white compartment of the CPP apparatus in the pre-conditioning and post-conditioning phases of saline and AMPH-treated female rats. Two-way RM ANOVA revealed a significant interaction [F (1, 22) = 9,595; *p* = 0.0053] and time [F (1, 22) = 7,871; *p* = 0.0103] effects, without significant treatment effect [F (1, 22) = 3,224; *p* = 0.0863]. According to the *post hoc* test, only animals injected with AMPH increased significantly (*p* < 0.001) the time spent in the white compartment associated with the drug during the post-conditioning phase. Figure 1C shows AVP tissue content in the LS of saline and AMPH-conditioned female rats. There was no significant difference in AVP content in the LS of AMPH-conditioned rats compared to saline-treated rats [U (38,133) = 17, *p* = 0.0831].

### 3.2 Experiment 2: effects of AVP microinjection in the LS on the expression of conditioning to AMPH in female rats

Figure 2 shows the effect of vasopressin microinjection intra-LS on the expression of AMPH conditioning place preference. Figure 2C shows that the bilateral AVP or AVP plus V_1A_ antagonist microinjection into the LS did not affect the locomotor activity of female rats [Two-way RM ANOVA: interaction (F (2, 33) = 05,471; *p* = 0.5838], treatment [F (2, 33) = 02,143; *p* = 08,083] and time effects [F (1, 33) = 02,201; *p* = 06,421].

Figure 2D shows the time the animal spent in the white compartment during the pre-conditioning and post-conditioning phases of the CPP protocol. On the post-conditioning day, females were microinjected with a vehicle, AVP, or AVP plus V_1A_ Ant intra-LS. Two-way RM ANOVA revealed a significant interaction [F (2, 38) = 8,967; *p* = 0.0006] and time effects [F (1, 38) = 19, 79; *p* < 0.0001], without treatment effect [F (2, 38) = 05,798; *p* = 0.5649]. According to the *post hoc* test, the animals microinjected with AVP did not show significant differences in the time spent at the white compartment between the pre-conditioning and the post-conditioning day (*p* = 0.8066). As expected, significant differences were observed in the vehicle group on the time spent in the white compartment between the pre-conditioning and the post-conditioning day (*p* < 0.001). Furthermore, to rule out the nonspecific effect of AVP, a specific V_1A_ receptor antagonist was co-microinjected. Like in the vehicle group, it was observed that the animals remained longer in the white chamber on the post-conditioning day (*p* < 0.01). Figure 2E shows representative heat maps (one animal in each condition) denoting the time spent in the three compartments of the CPP apparatus during the pre-conditioning and post-conditioning phases.

### 3.3 Experiment 3: effects of AVP on LS GABA and glutamate release in male and female rats

After showing that LS AVP microinjection impaired the expression of AMPH-induced CPP in both females and males (Gárate-Pérez et al., 2021), it was analyzed whether intra-LS perfusion of AVP modifies the extracellular levels of GABA and glutamate in the LS of male and female rats (Figure 3). Figures 3D, E show the effect of AVP perfusion on LS GABA and glutamate extracellular levels, respectively, in male and female rats. In males, AVP perfusion produced a significant increase in LS GABA extracellular levels [(Friedman = 30.24; *p* < 0.0001; 15 vs 75 (*p* = 0.0176), 15 vs. 90 (*p* = 0.0272), 15 vs. 105 (*p* = 0.0112), 30 vs. 105 (*p* = 0.0416), 45 vs. 75 (*p* = 0.0416) and 45 vs. 105 (*p* = 0.0416) min)]. However, in females, a decrease in GABA extracellular levels was observed in the LS at 90 (15 vs. 90: *p* = 0.0065) and 105 (15 vs. 105: *p* = 0.0175) min (Friedman = 21.50; *p* = 0.0015; Figure 3D). Regarding glutamate extracellular levels, no significant differences were observed during AVP perfusion in the LS, neither in males nor in females (Friedman = 5.082, *p* = 0.5334; Friedman = 8.143, *p* = 0.2278; Figure 3E). In the control group, no significant differences were observed after the change to a new one-aCSF solution, neither in GABA (Friedman = 4.286, *p* = 0.6381; Figure 3D) nor in glutamate (Friedman = 6.571, *p* = 0.3623; Figure 3E) extracellular levels.

### 3.4 Experiment 4: effects of AVP microinjection in the lateral septum on DA extracellular levels in the nucleus accumbens in female rats

Finally, to study if intra-LS microinjection of AVP modifies DA or GABA extracellular levels in the NAc in females after exposure to AMPH conditioning, microdialysis was performed in the NAc coupled with intra-LS microinjection (Figure 4). Figure 4D shows that microinjection of AVP into the LS did not change DA extracellular levels in the NAc of female rats [One-way ANOVA RM, treatment effects F (2,156, 8,624) = 1,739; *p* = 0.2322]. Similarly, no significant differences were observed in the NAc GABA extracellular levels after LS AVP microinjection (Friedman = 6.381; *p* = 0.2709) (Figure 4E).

## 4 Discussion

At present, the link between drugs of abuse and the extra hypothalamic vasopressinergic system in the context of the sexual dimorphism that encompasses its components has not been elucidated. This work proposed three objectives that attempted to tackle this problem. First, the effect of AMPH treatment on LS AVP content was studied in female rats, and the results showed that conditioning with AMPH did not change LS AVP content. We have previously reported in male rats that animals conditioned to AMPH showed decreased AVP levels in the LS (Gárate-Pérez et al., 2021), and in females, different treatments with AMPH did not change LS AVP levels (Ahumada et al., 2017). There is evidence of marked sexual dimorphism of the extra hypothalamic vasopressinergic system in the brain. However, little is known about sex differences in the effect of psychostimulants on the vasopressinergic system. It has been observed that acute administration of methamphetamine (10 mg/kg, i. p.), a psychostimulant with similar characteristics to AMPH, produced a time-dependent increase in AVP mRNA in the NAc, which was observed at week three and four post-injection (Jayanthi et al., 2018). In previous work, we found that, in females, AMPH sensitization increased the MeA and BNST AVP mRNA levels without changes in LS AVP content (Ahumada et al., 2017). Considering that both nuclei are the primary resources of LS AVP (de Vries, 2008), An increase in AVP release due to amphetamine treatment could explain why the observed increase in AVP mRNA does not translate into an increase in the protein levels in the LS (Ahumada et al., 2017). Several studies have shown that different psychostimulant treatments decrease AVP expression in the hypothalamus, suggesting an increase in the release of this neuropeptide (Godino and Renard, 2018).

Then, we wanted to study if there are sex differences in the effect of AVP administration in the LS upon the expression of conditioning to AMPH. A previous study showed that male rats conditioned to AMPH and microinjected with AVP intra-LS did not express the conditioning to AMPH (Gárate-Pérez et al., 2021). Here, we show that, in females, the AVP microinjection into the LS also decreased the expression of CPP to AMPH and that the V_1A_ receptor mediated this effect. Therefore, this effect is independent of the sex-associated differences in intra-LS AVP content in AMPH-conditioned animals and suggests the existence of some differentiation in the neurobiological mechanism underlying this behavior. Different concentrations of AVP might be necessary to elicit the same behavioral response in both sexes. In addition, studies have shown that DA release induced by AVP administration in the LS is different between males and females (Bredewold et al., 2018).

On the other hand, several publications have shown that intra-LS AVP administration has an anxiogenic effect (Liebsch et al., 1996; Everts and Koolhaas, 1999; Beiderbeck et al., 2007) and that the V_1A_ receptor mediates this effect (Landgraf et al., 1995). Besides, it has been suggested that high-anxiety rats are less sensitive to the rewarding effects of AMPH (Lehner et al., 2014). Therefore, it cannot be ruled out that the decreased time spent in the white compartment could be due to increased anxiety levels in females. Since we did not find differences in locomotor activity after AVP administration, we discarded alterations due to motor problems.

After observing that LS AVP administration abolished the expression of AMPH-induced CPP in both females and males, we decided to study the effect of AVP on LS GABA and glutamate release in male and female rats. Results showed that intra-LS perfusion of AVP increased extracellular GABA levels only in male rats. The V_1A_ receptor is coupled to a G_q/11_ protein subunit. Thus, the cellular effect induced by ligand binding to the receptor is to mobilize intracellular Ca^2+^, allowing the subsequent release of neurotransmitters (Ostrowski et al., 1992). In this sense, it has been reported that AVP can excite about 40% of the LS neuronal population through activation of the V_1A_ receptor (Raggenbass, 2008). Septal interneurons increased their inhibitory tone by activating the V_1A_ receptor in the presence of AVP. This response inhibited GABAergic projection neurons from the LS (Raggenbass, 2008). So, as we have previously suggested (Gárate-Pérez et al., 2021), in males, the inhibition of LS GABA projections disinhibited VTA GABA interneurons, decreasing DA release. However, LS AVP perfusion in females produced a different neurochemical mechanism that underlies the same behavior under the same experimental scheme. According to our results in females, it has been shown that the LS administration of V_1A_ receptor antagonists during social play, behavior that increases LS AVP release, increases GABA and glutamate extracellular levels (Bredewold et al., 2023). Although no differences have been observed in AVP receptor expression between males and females (Dumais and Veenema, 2016; Bredewold et al., 2023), there may be differences in the neurons and neuronal terminals that express the receptor between the sexes, which still need to be investigated.

The LS receives a robust glutamatergic transmission from the hippocampus (Sheehan et al., 2004), so it was decided to analyze the effect of intra-LS perfusion of AVP on glutamate levels in the LS. The depolarization of glutamatergic neurons reaching the LS (Luo et al., 2011) is usually followed by a hyperpolarization induced by the release of intraseptal GABA from the neurons, which maintains the inhibitory tone (Sheehan et al., 2004). Our results showed no changes in extracellular glutamate levels after intra-LS perfusion of AVP. This could be due, on the one hand, to the hyperpolarization of glutamatergic terminals produced by the increase in GABA induced by AVP; on the other hand, it could also be because the glutamatergic terminals that reach the LS do not express the V_1A_ receptor or the inhibitory tone on glutamatergic terminals outweighs the direct activation by AVP. Although it has been observed that the LS has a high expression of V_1A_ receptors (Ostrowski et al., 1992), their location at the presynaptic or postsynaptic level is not known.

Finally, to determine whether the behavioral effect observed in females correlates with DA release in the NAc after intra-LS AVP microinjection, we conditioned a group of females to AMPH. We measured NAc neurotransmitter extracellular levels after LS AVP microinjection. No significant differences in extracellular levels of DA or GABA in the NAc were observed. Previously, we demonstrated that, in males, LS AVP microinjection produced a significant decrease in DA in the NAc (Gárate-Pérez et al., 2021). Maybe, in females, LS AVP administration could be preventing the increase in NAc DA levels in a context associated with the drug and, in this way, decreasing the expression of AMPH-induced CPP. However, this hypothesis must be probed with further experiments. Peter and collaborators determined that after intracerebroventricular administration of oxytocin (OXT), voluntary consumption and preference for alcohol were reduced; they observed no significant changes in DA extracellular levels in the NAc, suggesting that OXT prevented the alcohol-induced increase in NAc DA levels after acute and repeated alcohol exposure in rats (Peters et al., 2017). On the other hand, it has been observed that after the systemic application of AMPH, the locomotor stimulant and rewarding effects of the drug are anatomically dissociated within the NAc. The core region contributes to motivated or goal-directed behavior, while the shell region contributes to the rewarding effects (Sellings and Clarke, 2003; Castro and Berridge, 2014). In addition, DA release in the different zones of the NAc varies according to the stimuli. Following a single injection of AMPH, there is a higher DA response in the core subregion than in the shell (McKitrick and Abercrombie, 2007). However, in response to novelty, DA efflux increased only in the shell and the shell-core transition zone (Rebec, 1998). Additionally, basal extracellular DA levels were higher in the core subregion than in the shell (McKitrick and Abercrombie, 2007; Hipólito et al., 2008). According to the placement of the microdialysis probe in our experiments, it is located in a region where it is impossible to precisely discriminate whether the measured extracellular DA levels originate from the shell or the core. Therefore, changes occurring in only one of these areas may have been masked by what happened in the other area. Further studies are needed to elucidate the underlying mechanism that explains the differences in neurochemical responses to AVP in male and female rats.

It is essential to mention that DA release is highly influenced by the reproductive cycle (Shimizu and Bray, 1993; Westwood, 2008). In this sense, the animals subjected to microdialysis to measure DA levels in the NAc were estrous and proestrus, stages in which estradiol levels are higher than diestrus (Sharma et al., 2020). Future studies will elucidate if, in other phases of the cycle, there is a decrease in DA release after AVP administration. Regarding behavioral studies, we previously showed no differences in the AMPH-locomotor sensitization protocol between females at different reproductive cycle stages (Ahumada et al., 2017). Therefore, we decided that our experimental conditioning place preference scheme would not consider the estrous cycle because this practice has been associated with increased animal stress levels (Sfikakis et al., 1996). In females, the vaginal smear procedure has been observed to generate stress and may decrease drug-induced activity (Papp et al., 1991; Walker et al., 2002). Therefore, our results are not restricted to one phase.

It is interesting to note that, despite observing no differences in AVP content in the LS in females conditioned to AMPH and not observing changes in GABA or glutamate levels in LS, behavioral solid responses associated with decreased expression of AMPH-induced CPP were observed in females microinjected with AVP in the LS. In this sense, it is reasonable to postulate that, in females, the decrease in conditioning generated by AVP microinjection is influenced by other factors inherent to gender, and an effect on anxiety cannot be discarded.

## Data Availability

The raw data supporting the conclusions of this article will be made available by the authors, without undue reservation.
